# Catabolism of l-rhamnose in *A. nidulans* proceeds via the non-phosphorylated pathway and is glucose repressed by a CreA-independent mechanism

**DOI:** 10.1186/s12934-020-01443-9

**Published:** 2020-10-02

**Authors:** Andrew P. MacCabe, Elpinickie I. Ninou, Ester Pardo, Margarita Orejas

**Affiliations:** 1 Instituto de Agroquímica y Tecnología de Alimentos (IATA), Consejo Superior de Investigaciones Científicas (CSIC), c/Catedrático Agustín Escardino Benlloch 7, 46980 Paterna, Valencia Spain; 2 Present Address: Center for Basic Research, Biomedical Research Foundation, Academy of Athens (BRFAA), 4 Soranou Ephessiou Street, 11527 Athens, Greece; 3 Present Address: ADM Biopolis, Parque Científico Universidad de Valencia, c/Catedrático Agustín Escardino Benlloch 9, 46980 Paterna, Valencia Spain

**Keywords:** *Aspergillus nidulans*, l-rhamnose catabolism, Transcriptional regulation, RhaR, CCR, CreA-independent, LRA, *lraA*/AN4186, l-rhamnose dehydrogenase, RT-qPCR, α-l-rhamnosidases

## Abstract

l-rhamnose (6-deoxy-mannose) occurs in nature mainly as a component of certain plant structural polysaccharides and bioactive metabolites but has also been found in some microorganisms and animals. The release of l-rhamnose from these substrates is catalysed by extracellular enzymes including α-l-rhamnosidases, the production of which is induced in its presence. The free sugar enters cells via specific uptake systems where it can be metabolized. Of two l-rhamnose catabolic pathways currently known in microorganisms a non-phosphorylated pathway has been identified in fungi and some bacteria but little is known of the regulatory mechanisms governing it in fungi. In this study two genes (*lraA* and *lraB*) are predicted to be involved in the catabolism of l-rhamnose, along with *lraC*, in the filamentous fungus *Aspergillus nidulans*. Transcription of all three is co-regulated with that of the genes encoding α-l-rhamnosidases, i.e. induction mediated by the l-rhamnose-responsive transcription factor RhaR and repression of induction in the presence of glucose via a CreA-independent mechanism. The participation of *lraA*/AN4186 (encoding l-rhamnose dehydrogenase) in l-rhamnose catabolism was revealed by the phenotypes of knock-out mutants and their complemented strains. *lraA* deletion negatively affects both growth on l-rhamnose and the synthesis of α-l-rhamnosidases, indicating not only the indispensability of this pathway for l-rhamnose utilization but also that a metabolite derived from this sugar is the true physiological inducer.

## Introduction

Metabolic versatility enables microorganisms to deal with fluctuating abiotic conditions and heterogeneous biological interactions, conferring the advantageous ability to utilise a diverse range of natural substrates as nutrients. To be able to metabolize complex organic polymers microorganisms must produce a variety of extracellular enzymes that catalyse polymer deconstruction into subunits that can be imported into the cell by transporter proteins. The subsequent catabolism of these soluble nutrients (e.g. sugars) occurs via metabolic pathways encoded by genes that may or may not be organised in clusters. Expression of an appropriate catabolic pathway can be critical for survival and microorganisms have developed sophisticated regulatory mechanisms to ensure the preferential use of those compounds that are most easily catabolized such as glucose; the use of alternative pathways is restricted to those conditions under which they are absolutely required (e.g. in the presence of a secondary carbon source when a preferred carbon source is not available).

Classified as a rare sugar due to its far lower abundance in nature compared to others, l-rhamnose (6-deoxy-l-mannose) occurs mainly as a component of certain plant structural polysaccharides though it has also been found in some microorganisms [1] and rarely in animal tissues [2]. In plants it is widely distributed in the primary cell wall pectic polysaccharides rhamnogalacturonan (RG) I and RGII (reviewed in [3–5]), in the seaweed polysaccharide ulvan (reviewed in [6, 7]), as well as in hemicelluloses, glycoproteins and diverse secondary metabolites (e.g. anthocyanins, flavonoids and terpenoids) ([8] and references therein). l-rhamnose can be used by many microbes as a nutrient and also acts as a signalling molecule provoking changes in gene expression. In this regard a number of microorganisms including certain yeasts and filamentous fungi have evolved l-rhamnose-inducible uptake and enzymatic systems that permit the utilization of this sugar as a sole carbon source when preferred carbon sources such as glucose are absent [9–13].

Two pathways have been described for the microbial assimilation of l-rhamnose (Additional file 1: Figure S1A). In many bacterial species such as *Escherichia coli* l-rhamnose is catabolized to dihydroxyacetone phosphate (DHAP) and l-lactaldehyde via the canonical phosphorylated pathway involving the products of the three genes *rhaA* (l-rhamnose isomerase)*, rhaB* (l-rhamnulose kinase) and *rhaC* (rhamnulose-1-P aldolase) ([9, 14, 15] and references therein). An alternative catabolic pathway involving non-phosphorylated intermediates has also been described in the Dothideomycete yeast-like fungus *Aureobasidium* (aka *Pullularia*) *pullulans* and the Saccharomycete yeasts *Scheffersomyces *(aka *Pichia*) *stipitis* and *Debaryomyces polymorphus* [16–18]. In the latter oxidative route l-rhamnose is enzymatically converted into pyruvate and lactaldehyde by four consecutive reactions catalysed by l-rhamnose-1-dehydrogenase (LRA1), l-rhamnono-γ-lactonase (LRA2), l-rhamnonate dehydratase (LRA3) and l-2-keto-3-deoxyrhamnonate (L-KDR) aldolase (LRA4). The four enzymes of *S. stipitis* have been shown to be encoded in a gene cluster named *LRA* (Additional file 1: Figure S1B) and conservation or partial conservation of this cluster is apparent in other l-rhamnose utilizing yeasts (e.g.* Candida lusitaniae*), in various filamentous fungi (e.g.* Fusarium graminearum*, *Aspergillus fumigatus* and *Cryptococcus neoformans*) and in some bacteria such as *Azotobacter vinelandii* and *Burkholderia cenocepacia* [10, 11]. Modified versions of these two catabolic pathways have also been identified in bacteria [9, 19]. Whilst *LRA1*-*LRA4* in *S. stipitis, LRA4* in *Pichia pastoris* and the first three catabolic genes *lraA, lraB* and *lraC* (clustered) of *Aspergillus niger* have been functionally characterized [10, 11, 20–22], knowledge of the regulatory mechanisms controlling l-rhamnose catabolism in fungi is sparse, especially carbon catabolite repression (CCR) induced by glucose. In *A. pullulans* and *S. stipitis* it has been reported that l-rhamnose-1-dehydrogenase activity is induced by l-rhamnose and that this induction is repressed by d-glucose [17, 23, 24] but the corresponding transcription factors are unknown.

Similarly to other fungi, *A. nidulans* is able to grow on l-rhamnose as a sole carbon source [25]. Homology searches have identified genomically scattered orthologous sequences potentially encoding the first three steps of the non-phosphorylated catabolic pathway (the current work; Additional file 1: Figure S1B) but to date none has been functionally characterized in this fungus. Thus their involvement in the assimilation of this sugar and the function of the non-phosphorylated pathway in *A. nidulans* have yet to be formally demonstrated. We have previously shown that deletion of the regulatory gene *rhaR* (encodes the Zn_2_Cys_6_ l-rhamnose-responsive transcription activator RhaR) in both *A. nidulans* and *Neurospora crassa* (AN5673 and NCU09033, respectively) negatively affects the ability of these fungi to grow on l-rhamnose as a sole carbon source, and in *A. nidulans* this may be contributed at least in part through its influence on the expression of *lraC* [26]. Although *rhaR* is divergently transcribed from the adjacent *lraC* gene in *A. nidulans* (and other filamentous fungi; Additional file 1: Figure S1B), *rhaR* and *lraC* are not co-regulated: whereas expression of *rhaR* is constitutive that of *lraC* is induced when l-rhamnose is present as sole carbon source and this is mediated by RhaR [26]. In the presence of l-rhamnose RhaR also induces the expression of the *A. nidulans* genes *rhaA* and *rhaE* (encoding two GH78 α-l-rhamnosidases that provide the inducing carbon source) which are thus co-expressed with *lraC* [26]. Whether other *lra* genes are subject to induction by RhaR in the presence of l-rhamnose is not known. Nor is it known whether the l-rhamnose catabolic pathway is repressed by glucose and if so what role if any is played by the wide domain repressor CreA, the only carbon catabolite repressor protein known to date in filamentous fungi. In regard to the later, we have previously shown that, unlike many other glycoside hydrolase genes, *rhaA* and *rhaE* are repressed by glucose and other carbon sources in a manner independent of CreA. In addition, studies on mixed carbon sources indicated that inducer exclusion may play a prominent role in α-l-rhamnosidase gene regulation [12].

The characterization of catabolic genes and especially knowledge of their regulation can provide a conceptual framework upon which strategies can be designed to adjust the flux of inducers and repressors into the fungal cell in order to modulate the productivity of fungal cell factories. The present study examines the roles played by l-rhamnose and d-glucose in controlling processes related to the metabolism of l-rhamnose in *A. nidulans*. Specifically, we addressed the following points: (1) to establish whether *A. nidulans* catabolizes l-rhamnose via the non-phosphorylated/oxidative LRA pathway, (2) to characterize a gene from this pathway, demonstrating its function and determining whether l-rhamnose catabolism occurs exclusively via this route, (3) to determine whether l-rhamnose or a product of its catabolism is the intracellular signalling molecule that induces gene expression, and (4) to establish whether glucose represses the *lra* genes and the role played by CreA if any.

## Materials and methods

### Strains, media and growth conditions

*Escherichia coli* strain DH5α (*sup*E44, ΔU169 (ϕ80*lac*ZΔM15, *hsd*R17, *rec*A1, *end*A1, *gyr*A96, *thi*-1, *relA*1)) was used as the host for cloning experiments and plasmid amplification. Fungal strains used in this study are listed in Table 1. Non-homologous end-joining (NHEJ)-deficient *A. nidulans* strain AR198 (*argB2*; *pyroA4*, Δ*nkuA*::*argB*; *riboB2*; AKA TN02A21, [27]) was used to delete *lraA*/AN4186. AR271 (*argB2*; *pyroA4*, Δ*nkuA*::*argB*; *riboB2*::Af*_riboB*; [26]) was used as a riboflavin prototrophic (nutritional) control strain isogenic to gene deletion mutants generated in AR198. *A. nidulans* wild type AR5 (*biA1*) and AR305 (*creA*^*d*^*30, biA1*) strains were used for expression analyses.

**Table 1:** *Aspergillus nidulans* strains used in this study

Strain code	Name	Genotype^a^	References
AR5	*biA1*	*biA1*	M. A. Peñalva (CIB/CSIC)
AR305	*creA*^*d*^*30*	*biA1, creA*^*d*^*30*	M. A. Peñalva/ E. Espeso (CIB/CSIC)
AR198	TN02A21	Δ*nkuA::argB, argB2*, *riboB2, pyroA4*	[27]
AR225	Δ*rhaR*	Δ*nkuA::argB, argB2*, *riboB2, ΔrhaR::*Af_*riboB, pyroA4*	[26]
AR271		Δ*nkuA*::*argB, argB2*, *riboB2*::Af_*riboB, pyroA4*	[26]
AR274	FGSC A4		[28]
AR245		Δ*nkuA::argB, argB2*, Δ*lraA*::Af_*riboB*, *riboB2, pyroA4*	This work
AR246		Δ*nkuA::argB, argB2*, Δ*lraA*::Af_*riboB*, *riboB2, pyroA4*	This work
AR247		Δ*nkuA::argB, argB2*, Δ*lraA*::Af_*riboB*, *riboB2, pyroA4*	This work
AR248		Δ*nkuA::argB, argB2*, Δ*lraA*::Af_*riboB*, *riboB2, pyroA4*	This work
AR501		Δ*nkuA::argB, argB2*, Δ*lraA*::Af_*riboB*, *riboB2,* Δ*pyroA4::lraA-Af_pyroA*	This work
AR502		Δ*nkuA::argB, argB2*, Δ*lraA*::Af_*riboB*, *riboB2,* Δ*pyroA4::lraA-Af_pyroA*	This work
AR503		Δ*nkuA::argB, argB2*, Δ*lraA*::Af_*riboB*, *riboB2,* Δ*pyroA4::lraA-Af_pyroA*	This work
AR504		Δ*nkuA::argB, argB2*, Δ*lraA*::Af_*riboB*, *riboB2,* Δ*pyroA4::lraA-Af_pyroA*	This work

^a^With the exception of AR274 all the *A. nidulans* strains carry the mutant allele *veA1*

*E. coli* was grown in Luria–Bertani (LB) medium (1% w/v tryptone, 0.5% w/v yeast extract, 1% w/v NaCl) with 100 μg/ml ampicillin when selection was required. *A. nidulans* strains were grown in either minimal (MM), MM + 0.5% w/v yeast extract (YMM) or complete (CM) media [28, 29] containing 1% w/v carbon source, unless specified otherwise, and supplemented with the appropriate requirements. Carbon sources were added from filter-sterilised concentrated stocks. Ammonium tartrate (5 mM) was used as the nitrogen source unless stated otherwise. For solid media 1.5% w/v agar was added.

For the evaluation of α-l-rhamnosidase and l-rhamnose dehydrogenase activities in transfer experiments, mycelial biomass was generated from an inoculation yielding a final titre of 5 × 10^6^ spores/ml in YMM to which 2% lactose was added as the sole carbon source. Growth was conducted for 18 h at 37 °C with orbital shaking at 180–200 rpm in Erlenmeyer flasks respecting a 5:1 ratio between the total flask volume and the volume of the liquid culture. Mycelia were harvested, washed with MM lacking a carbon source and transferred (~ 1 g drained wet weight) to 100 ml Erlenmeyer flasks containing 20 ml of induction medium (YMM + 1% l-rhamnose as the sole carbon source). Lactose was chosen for initial growth of mycelia since previous studies in our group established it to be the least repressive carbon source regarding rhamnosidase synthesis and rhamnosidase gene induction in *A. nidulans* [12, 25].

For RNA extraction in transfer experiments, mycelial biomass was generated from spores inoculated (5 × 10^6^ spores/ml) into YMM + 5 mM urea (nitrogen source) to which 0.1% fructose was added as the sole carbon source. After 18 h of growth as described above, mycelia were harvested, washed with MM lacking a carbon source and transferred (~ 1.5 g) to induction medium (MM + urea; 15 ml) for 3 h in which fructose was substituted by l-rhamnose at 1%; inducing-repressing medium substituted fructose with 1% l-rhamnose + 1% glucose.

### Transformation

*A. nidulans* transformation of the NHEJ-deficient (Δ*nkuA*) strain AR198 was undertaken based on the methods of Tilburn et al. [30] and Szewczyk et al. [31]. Protoplasts were generated using Vinoflow FCE lysing enzyme (Novozymes) or Lysing Enzymes from *Trichoderma harzianum* (Sigma).

### Nucleic acid procedures

Standard molecular techniques were as described in Sambrook and Russell [32]. PCR reactions were performed using Phusion High-Fidelity (Thermo Fisher) or Top-Taq (BIORON) DNA polymerases. Restriction and modifying enzymes were sourced from Roche Diagnostics, New England BioLabs and Thermo Fisher. All products were used as recommended by the manufacturer. DNA sequencing was carried out using the BigDye Terminator v3.1 Cycle Sequencing Kit and run on an ABI PRISM 310 Genetic Analyser (Applied Biosystems, USA) in the Central Service for Experimental Research (SCSIE) of the University of Valencia. Chromatograms were analysed using the programs Chromas 2.6.5 or SnapGene Viewer 4.1.9. Genomic DNA extracted from strains AR198 and FGSC A4 (named AR274 in our collection) were used as templates for the isolation of *A. nidulans* fragments by high-fidelity PCR. Oligonucleotide primers used in this study are listed in Additional file 2: Table S1.

For the preparation of total RNA from transfer cultures, mycelial mass was recovered by filtration through Nytal mesh, rapidly pressed dry between sheets of absorbent paper and flash-frozen in liquid nitrogen; if not used immediately the pressed mycelium was stored at − 80 °C. Approximately 200 mg of frozen mycelium was placed in a 2 ml screw-capped tube containing 850 μl RNA Plus (MP Biomedicals, USA) and 5 stainless steel homogenisation beads of 2.3 mm diameter (BioSpec, USA) and homogenised immediately in a mini-Beadbeater (BioSpec) for 10 s at 4200 rpm. Homogenisation was repeated three times more with cooling intervals on ice. The protocol provided with RNA Plus was followed and the resulting isopropanol-pelleted material was washed with 70% ethanol and further purified as follows: the pellet was dissolved in 500 μl DEPC-treated milliQ water, mixed with three volumes of 4 M sodium acetate (pH 6) and stored at − 20 °C for 1–2 h; RNA was recovered by centrifugation (12,000*g* for 20 min at 4 °C), dissolved in DEPC-treated milliQ water and re-precipitated with sodium acetate and ethanol; after recovery by centrifugation and washing with 70% ethanol the pellet was completely dissolved in 750 μl DEPC-treated milliQ water and 250 μl of 10 M lithium chloride solution was then added; after mixing, the sample was incubated for at least 1 h at − 20 °C and RNA was subsequently recovered by centrifugation; the final pellet was again dissolved in DEPC-treated milliQ water, re-precipitated with sodium acetate and ethanol, washed with 70% ethanol and finally dissolved in 100 μl DEPC-treated milliQ water. RNA concentration and purity were assessed using a NanoDrop ND-1000 Spectrophotometer (Nano-Drop Technologies) and RNA integrity was verified by agarose gel electrophoresis.

### RNA sequencing (RNA-Seq)

cDNA library construction (TruSeq Stranded mRNA kit—Illumina) and sequencing (single-end 1 × 75 bp; Illumina Nextseq 550) were performed by the Central Service for Experimental Research (SCSIE) of the University of Valencia using total RNA as starting material. Prior to its use, the purity and integrity of the total RNA was assessed using a 2100 Bioanalyser (Agilent) and quantified by Qubit fluorometry (ThermoFisher). The TruSeq kit was used following the manufacturer's instructions. Read quality was checked using FastQC v0.11.3 (https://www.bioinformatics.babraham.ac.uk/projects/fastqc/) both before and after pre-processing. Raw data were pre-processed with Cutadapt v1.8.3 [33] to remove adapter sequences, filter reads of < 20 nt and trim 3′ ends using a base quality threshold of 28. Reads were mapped to the *A. nidulans* genome (AspGD version s10-m04-r16) using Tophat v2.1.0 [34]. Raw data quantification was done with Seqmonk v1.42 (https://www.bioinformatics.babraham.ac.uk/projects/seqmonk/) and expressed as reads per million mapped reads (RPM). Within the SARTools R package [35], DESeq2 v1.18.1 [36] was used for differential expression analysis of raw counts; the false discovery rate (FDR) was obtained from *p*-values using the Benjamini–Hochberg procedure. Only *p*-values below 0.05 were used to identify significant differences in gene expression.

### Semi-quantitative reverse-transcriptase PCR (RT-sqPCR) and quantitative PCR (RT-qPCR)

To prepare template material for RT-qPCR, 20 μg of total RNA was treated with 1 μl (10 U) RNase-free DNaseI (Roche) in the presence of 0.5 μl (20 U) RNaseOUT (Invitrogen) in a final volume of 50 μl at 37 °C for 30 min. DNase activity was inactivated at 75 °C for 10 min followed by snap chilling on ice for 5 min. After a very brief spin, 5 μl of this preparation was added to a PCR tube along with 6 μl DEPC-treated milliQ water, 1 μl of oligo (dT)_12–18_ primer (0.5 μg/μl) and 1 μl of dNTPs (10 mM each). This mixture was incubated at 65 °C for 5 min and then snap chilled on ice. After a very brief spin, the following components were added and mixed and the whole was incubated for 1 h at 50 °C: 4 μl 4 × first-strand buffer (Invitrogen), 1 μl DTT (0.1 M), 1 μl (40U) RNaseOUT and 1 μl (200U) Superscript III reverse transcriptase (Invitrogen). Superscript was inactivated by incubation at 70 °C for 15 min. Amplification of cDNA targets was performed using an aliquot (2 μl; 1/10th) of the RT reaction and specific primers in final volumes of 10 μl.

RT-sqPCR: sense and antisense oligonucleotide primers were located either side of intron/exon boundaries to differentiate genomic and cDNA sequences. The actin gene *actA* (AN6542) was used as a loading control. To confirm that PCR amplifications were in a linear range, cycle titrations were performed for each gene as described previously [26]. From these analyses, 24 cycles were determined to be optimal for *actA* (primers 97 and 98) and 25 cycles for *lraA*/AN4186 (primers 120 and 121) in the AR5 (*biA1*) wild type strain. The data were confirmed in two independent experiments.

RT-qPCR: reactions were performed using SYBR Green PCR reagents (Roche) in a LightCycler 480 instrument (Roche). Three biological replicates were undertaken for each biological condition and qPCR of each was performed in triplicate (technical replicates)—these procedures were conducted at different times and by more than one operator. Cycling conditions were: 95 °C for 5 min, followed by 45 amplification cycles of 10 s at 95 °C, 10 s at 60 °C, 12 s at 72 °C; for product melting data a single cycle of 1 min at 65 °C followed by a linear increase in temperature to 95 °C at a rate of 0.11 °C/s completed the run. Cycle threshold (Ct) data were obtained using the LightCycler 480 software package v1.5.0.39. The genes encoding histone H2B/AN3469 (primers 394 and 395) and beta-tubulin *benA*/AN1182 (primers 386 and 387) were used as references. The stability of expression of these genes in our biological samples was verified using geNorm [37]. Primers for RT-qPCR were designed with the help of the Primer-BLAST tool (NCBI) and were chosen to cross intron/exon junctions where feasible. The specificity of primer pair products was confirmed by gel electrophoresis where possible, and melting curve analysis was carried out in all cases. Primer pair efficiencies where obtained by qPCR (values are given in Additional file 2: Table S1). Relative quantification of reference-gene-normalized target genes was determined using the Relative Expression Software Tool (Multiple Condition Solver REST-MCS v2) [38] and REST 2009 (Qiagen).

### Generation of *lraA* disruption mutants and complemented strains

In order to generate an *A. nidulans* AN4186 null mutant a deletion cassette was constructed in pBluescript SK ( +) (Stratagene). gDNA sequences flanking the AN4186 CDS were obtained as PCR-generated fragments: oligonucleotide pair LRA1_NotI (61) and LRA1_rev (62), which incorporate *Not*I and *Eco*RI restriction sites respectively, were used to amplify the 5′-UTR (1779 bp) region; oligonucleotide pair LRA1_dir (63) and LRA1_KpnI (64), which incorporate *Eco*RI and *Not*I-*Kpn*I restriction sites respectively, amplified the 3′-UTR (1514 bp). These PCR fragments were cloned into the *Not*I and *Kpn*I sites of pBluescript SK ( +) yielding plasmid pE406 and the absence of mutations was confirmed by sequencing (oligonucleotides 141 and 142). A 1.9 kb *Eco*RI fragment comprising the *A. fumigatus riboB* (Afu1g13300) expression cassette (Af_*riboB*, obtained from plasmid pTN2 [27]) was then subcloned into the *Eco*RI site of pE406 to generate pE407. Deletion of AN4186 was achieved by transforming protoplasts of strain AR198 with the *Not*I deletion cassette (5.3 kb) isolated from pE407 and selecting for transformants able to grow in the absence of riboflavin. Riboflavin prototrophs were purified, tested for growth on l-rhamnose and analysed by diagnostic PCR and Southern blot analysis.

To complement the AN4186 deletion, a DNA fragment comprising the wild type AN4186 locus including sequences upstream and downstream of the CDS (804 and 623 bp respectively) was generated by high-fidelity PCR from gDNA of strain AR198 using the oligonucleotide pair 547–548 and cloned into the *Sma*I site of pVAL159 yielding plasmid pE528 (pVAL159 comprises the *A. fumigatus pyroA* cassette from pTN1 [27] flanked by the *A. nidulans pyroA*/AN7725 upstream and downstream sequences (UTR) cloned in pBluescript SK ( +)). A 5.8 kb fragment obtained by high-fidelity PCR from pE528 using primers 602 and 603 comprising four DNA sequence elements (5′UTR *pyroA*/AN7725–*lraA*-Af_*pyroA*-3′UTR *pyroA*/AN7725) was used to transform Δ*lraA* strain AR247 and ectopically express *lraA* at the AN7725 locus. Transformants C1–C4 (AR501-AR504) were selected for growth in the absence of pyridoxine, and the integrities of the *lra*A complementing cassette as well as the original *lraA* disruption cassette present in AR247 were confirmed by PCR (see “Results and discussion”).

### α-l-Rhamnosidase assays

α-l-Rhamnosidase activity in cell-free extracts was measured using the artificial substrate *p*-nitrophenyl-α-l-rhamnopyranoside (*p*NPR). The release of *p*-nitrophenol was measured spectrophotometrically at 400 nm. The assay was performed in 96-well microtitre plates in final volumes of 250 μl using 1.4 mM substrate in 100 mM McIlvaine buffer (citrate–phosphate buffer) pH 4.0 and incubated at 50 °C for 15 min with shaking essentially as described previously [39]. To assess the α-l-rhamnosidase activity of *A. nidulans* colonies in vivo, MM plates were supplemented with 40 μM 4-methylumbelliferyl α-l-rhamnopyranoside (MUR) and 1 mM McIlvaine buffer pH 4.0 as described [12]. Cell-free extracts were from duplicate growths and rhamnosidase assays were performed in duplicate.

### l-Rhamnose dehydrogenase activity

l-Rhamnose dehydrogenase activity was measured by detecting the formation of NADH as described previously [20] in crude cell-free extracts obtained by sonication (1 pulse of 1 min and 5 pulses of 30 s, with resting periods in ice) and subsequent centrifugation at 4 °C. Reactions were carried out at 37 °C for 6 h in final volumes of 200 μl comprising 45 μl Tris–HCl 100 mM (pH 8.0), 50 μl Tris–HCl 200 mM (pH 8.0), 10 μl distilled water, 40 μl NAD 2 mM, 5 μl extract (≡10 μg total protein) and 50 μl 50 mM l-rhamnose (the latter was substituted by 50 μl Tris–HCl 100 mM for the control). A Polarstar Omega microplate reader (BMG LabTech) was used to measure and analyse the data. The increase in absorbance at 340 nm was used to monitor the formation of NADH. Protein concentrations were measured by the Bradford method using lysozyme as standard [40].

## Results and discussion

### In silico evidence for the non-phosphorylated l-rhamnose catabolic pathway in *A. nidulans*

In most bacteria l-rhamnose is converted to l-rhamnulose by l-rhamnose isomerase (EC 5.3.1.14; RhaA [14]) in the first reaction of the canonical phosphorylated pathway. By contrast, in some other bacteria and fungi l-rhamnose is metabolised to l-rhamnono-γ-lactone by l-rhamnose 1-dehydrogenase (EC 1.1.1.173) which is encoded by the *LRA1/RHA1* gene (Additional file 1: Figure S1A) [11, 20]. BlastP searches of the *A. nidulans* genome (AspGD; https://www.aspergillusgenome.org/) failed to identify homologues of the *E. coli* protein RhaA, strongly suggesting the existence of an alternative pathway for the catabolism of l-rhamnose. Accordingly, a BlastP search using the *S. stipitis* Lra1/Rha1 protein (jgi|Picst3|50944) as the query sequence identified the protein encoded by locus AN4186 (Table 2) as yielding the best bidirectional hit (271 amino acids (aa) in length; E-value 5e−86; 63.5% identity). AN4186 is currently annotated as encoding a putative glucose 1-dehydrogenase (GudB) and is located on chromosome II. The next closest hit was AN1886 (246 aa, E-value 5e−61 and 55.6% identity) which is annotated as being a putative tetrahydroxynaphthalene reductase.

**Table 2:** Identification and expression (RPKM) of *A. nidulans* genes homologous to the LRA genes of *S. stipitis*

Probe (*S. stipitis*)	*A. nidulans* locus (AspGD)	E value (< 1E−30)	Reverse Blast. Hit of lowest E value (< 1e-30)	RPKM: AR271 on lactose	RPKM: AR271 on rhamnose	RPKM: AR225 on rhamnose
LRA1/RHA1 (jgi|Picst3|50944)	AN4186 (chr. II)	5.0E−86	Picst3|50944 (1.24E−90)	11	983	9
	AN1886 (chr. VII)	5.0E−61	Picst3|50944 (1.06E−53)	0.31^a^	0.49^a^	0.45^a^
LRA2 (jgi|Picst3|63908)	AN3740 (chr. II)	5.0E−56	Picst3|63908 (5.18E−42)	28	185	35
LRA3 (jgi|Picst3|50672)	AN5672 (chr. V)	5.0E−171	Picst3|50672 (0)	49	1785	116
LRA4 (jgi|Picst3|64442)	AN7929 (chr. II)	9.0E−40	Picst3:64442 (3.1E−35)	0^a^	0.57^a^	3
	AN0617 (chr. VIII)	1.0E−38	Picst3:64442 (2.47E−30)	0.78^a^	1.27^a^	1.43^a^
	AN10990 (chr. IV)	1.0E−35	Picst3:64442	6	6	3
	AN2859 (chr. VI)	1.0E−33	Picst3:64442	30	28	33
	AN1503 (chr. VII)	2.0E−33	Picst3:64442	55	23	44
	Rhamnosidases:					
	*rhaA*/AN10277			0.55^a^	1244	3
	*rhaE*/AN7151			0.19^a^	542	0.4^a^
	Reference genes:					
	*nkuA*/AN7753			1.21^a^	1.74^a^	1.57^a^
	*benA*/AN1182			207	246	226
	H2B/AN3469			2490	2888	2642

Transcript abundances (RPKM, i.e. reads per kilobase of exon model per million mapped reads) are averages of triplicates. *A. nidulans* strain AR225 is deleted for the transcription activator gene *rhaR* and AR271 is the corresponding nutritional isogenic *rhaR*^+^ control

^a^Considered as not expressed

RNA-Seq analyses (our work to be published in detail elsewhere) have shown AN4186 transcript abundance (reads per kilobase million—RPKM) to be significantly greater (983 *vs* 11; DEseq2 *p*-value 1.6E−67) in *A. nidulans* strain AR271 transferred to medium containing l-rhamnose as sole carbon source compared to transfers of the same strain to medium containing lactose, thus indicating specific induction of that locus on l-rhamnose (Table 2). Under inducing conditions (l-rhamnose) AN4186 RPKM were significantly lower in the strain (AR225) deleted for the transcription factor *rhaR/*AN5673 compared to AR271 (9 *vs* 983; DEseq2 *p*-value 9.6E−74—Table 2). By contrast, no expression (RKPM) was observed for locus AN1886 under the conditions studied. Taken together these data strongly suggest that AN4186 (named *lraA*) is the functional homologue of *LRA1/RHA1*. An *lraA* cDNA (ATG to STOP) was obtained by high-fidelity PCR using the oligonucleotide pair 120 and 122 and subcloned into the *Eco*RV site of pBluescript SK ( +). The insert (886 bp), present in two independently obtained plasmids pE462 and pE463, was sequenced (oligonucleotides 141 and 142) revealing *lraA* to be interrupted by three consensus (GT-AG) introns of 90, 67 and 50 nucleotides thus confirming its predicted intron/exon structure (AspGD).

In the *S. stipitis* LRA pathway l-rhamnono-γ-lactone (i.e. the product of the reaction catalysed by Lra1/Rha1) is further metabolized to l-rhamnonate and subsequently to l-2-keto-3-deoxyrhamnonate (L-KDR) in two consecutive reactions catalysed by l-rhamnono-γ-lactonase (Lra2; EC 3.1.1.65) and l-rhamnonate dehydratase (Lra3; EC 4.2.1.90) respectively (Additional file 1: Figure S1A). Bidirectional BlastP analyses resulted in the identification of a unique homologue candidate for each enzyme among the repertoire of hypothetical *A. nidulans* proteins: AN3740 (360 aa; E-value 5e−56; 34.7% identity to Lra2; maps to chromosome II but is located far from AN4186) and AN5672 (425 aa; E-value 5e−171; 66.5% identity to Lra3; chromosome V). RNA-Seq analyses showed co-expression (i.e. specific induction on l-rhamnose mediated by RhaR) of these two loci with those of AN4186/*lraA* and the α-l-rhamnosidase genes *rhaA* and *rhaE* (Table 2). Moreover, as noted previously [26] AN5672 is adjacent to and divergently transcribed from the *rhaR* (AN5673) regulatory gene, a topology maintained in certain other filamentous ascomycetes (e.g. *Neurospora crassa*). These data strongly suggest that AN3740 (named *lraB*) along with AN5672 (named *lraC*) are the functional homologues of *LRA2* and *LRA3* respectively.

The fourth step in the LRA pathway is the aldolase-mediated conversion of L-KDR to pyruvate and l-lactaldehyde. To date, eukaryotic/fungal L-KDR aldolases have only been identified in the related yeasts *S. stipitis* and *P. pastoris* [11, 21]. Bidirectional BlastP analyses combined with gene expression and gene deletion studies failed to identify the functional homologue of *LRA4* in *A. niger* [22]. Our BlastP screening of the *A. nidulans* proteome also failed to reveal a clear *LRA4* candidate, yielding instead five low-similarity targets (AN7929 E-value 9e−40, AN0617 1e−38, AN10990 1e−35, AN2859 1e−33 and AN1503 2e−33). The potential physiological relevance of these loci was assessed by comparing their expression (RPKM) in mycelia transferred to medium containing l-rhamnose with a reference medium containing lactose. Expression of the five *loci* was either absent or not co-regulated with *lraA*-*lraC* or the rhamnosidases (Table 2).

From the above, *A. nidulans* genes potentially encoding l-rhamnose catabolic activities have been identified based on sequence homologies with the previously characterized *LRA1*-*LRA3* genes of *S. stipitis* [11]. RNA-Seq has shown the expression of loci AN4186, AN3740 and AN5672 to coincide with that of the α-l-rhamnosidase genes *rhaA* and *rhaE*, findings that concord with studies in *A. niger* [22, 41] and *N. crassa* [42]. A challenge for our future studies will be the identification of the *A. nidulans* gene encoding the catalytic step corresponding to LRA4. With regard to the latter, AN7929, AN2859 and AN1503 are orthologues of the *lraD1*, *lraD2* and *lraD3 loci* in *A. niger*, the deletions of each of which failed to affect the ability of *A. niger* to grow on l-rhamnose [22].

### Deletion of *lraA*/AN4186 severely impairs l-rhamnose utilization

**Fig. 1:**
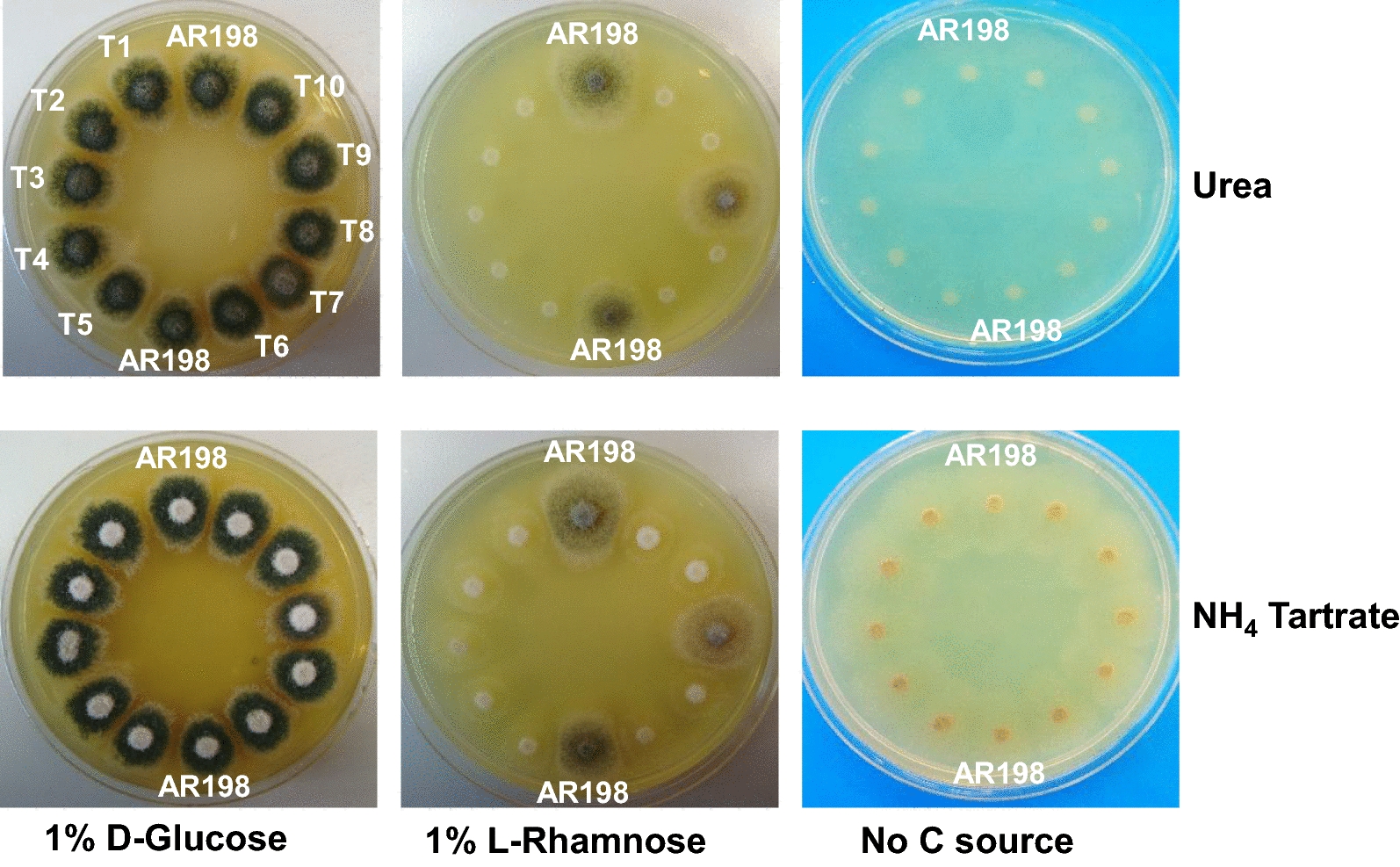
Deletion of *lraA*/AN4186 leads to reduced growth on l-rhamnose. The untransformed strain (AR198) and riboflavin prototrophic transformants (T1 to T10) were grown (3 days at 37 °C) on minimal media supplemented with riboflavin (required by AR198) and pyridoxine (required by all strains) containing different carbon and nitrogen sources. Nine transformants show considerably impaired growth on l-rhamnose. 1.5 × 10^5^ conidia were spotted in 3 μl drops

**Fig. 2:**
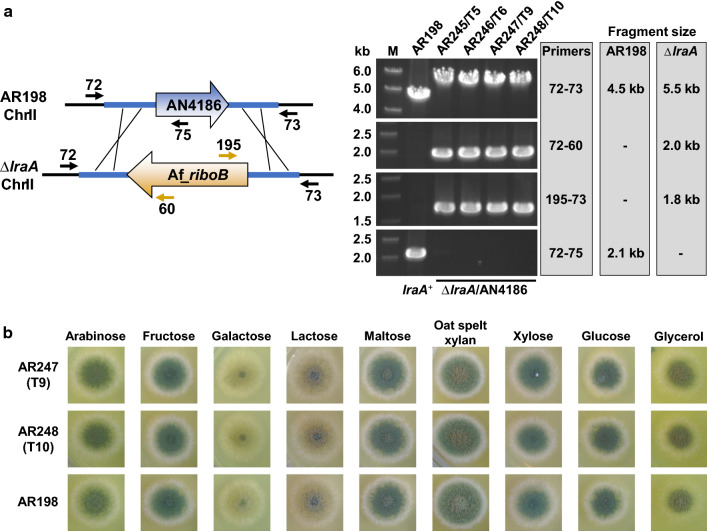
Identification, gene replacement analysis and growth tests of *A. nidulans* Δ*lraA* mutant strains. **a** Schematic diagram of the *lraA*/AN4186 locus in AR198 and the gene replacement event in Δ*lraA* strains. Correct replacement of *lraA*/AN4186 with the Af_*riboB* expression cassette in four selected transformants (T5, T6, T9 and T10) was verified by the absence or appearance of PCR products of the expected sizes using CDS-located primers (60, 75 and 195) along with primers 72 and 73 located outside the *lraA* flanking sequences used in the gene replacement cassette. **b** *A. nidulans* Δ*lraA* mutant (AR247 and AR248) and untransformed (AR198) strains grown (3 days at 37 °C) on solid MM containing different carbon sources (1%) and supplemented with pyridoxine and riboflavin. Equal numbers of conidia (10^4^) were spotted (2 µl) from a sterile suspension in 0.005% Tween 80. Complete genotypes are given in Table 1

To test whether the predicted LRA pathway is necessary and sufficient for growth on l-rhamnose, targeted deletion of the candidate gene for the first catabolic step (*lraA*/AN4186) was achieved by replacing its open reading frame (from − 17 to − 7 relative to the ATG and stop codons respectively) in AR198 with the *A. fumigatus riboB* expression cassette (Af_*riboB*) that can complement the *A. nidulans riboB2* mutant allele (the mutant allele is deleted for a single cytosine residue (CTT.CGT.CAA… to CTT.gtc.aag…) resulting in a frameshift after Leu285 and protein truncation after the addition of 145 novel amino acids; [26]). The phenotypes of ten clonally purified riboflavin prototrophic transformants together with that of the parental strain AR198 were first assessed on appropriately supplemented MM plates containing either glucose or l-rhamnose as sole carbon source in the presence of either urea (a poor N source but not a carbon source [43]) or ammonium tartrate (a preferred N source that can also be used as a poor carbon source). Nine of the transformants showed the same growth as the parental strain on glucose medium. Their growth on l-rhamnose however was severely affected, the mycelial sparsity observed resembling that seen both on control plates lacking sugar (Fig. 1) and *rhaR* loss-of-function mutants on rhamnose [26]. Similar consequences for rhamnose utilization have also been reported in *A. niger* [22]. To confirm integration of the disruption cassette at the AN4186 locus, four transformants (T5, T6, T9 and T10, renamed AR245-AR248 respectively) were selected and analysed by diagnostic PCR. Figure 2a shows oligonucleotide pairs 72–73, 72–60 and 195–73 yielded fragments of 5.5, 2.0 and 1.8 kb in all four as predicted for replacement of *lraA* by the *Af_riboB* cassette whereas fragments of 4.5 kb and 2.1 kb were obtained for the parental strain AR198 with oligo pairs 72–73 and 72–75. Southern blot analysis was consistent with single copy integrations at the homologous locus and the absence of ectopic integrations. Given the identical nature of the phenotype and results of the molecular analyses for the four transformants, AR247 and AR248 were randomly chosen for further work. Growth tests on a range of other carbon sources showed similar growth of these transformants and the control thereby corroborating the specific role of *lraA* in the utilization of l-rhamnose (Fig. 2b).

### Enzymatic analysis of LraA

**Fig. 3:**
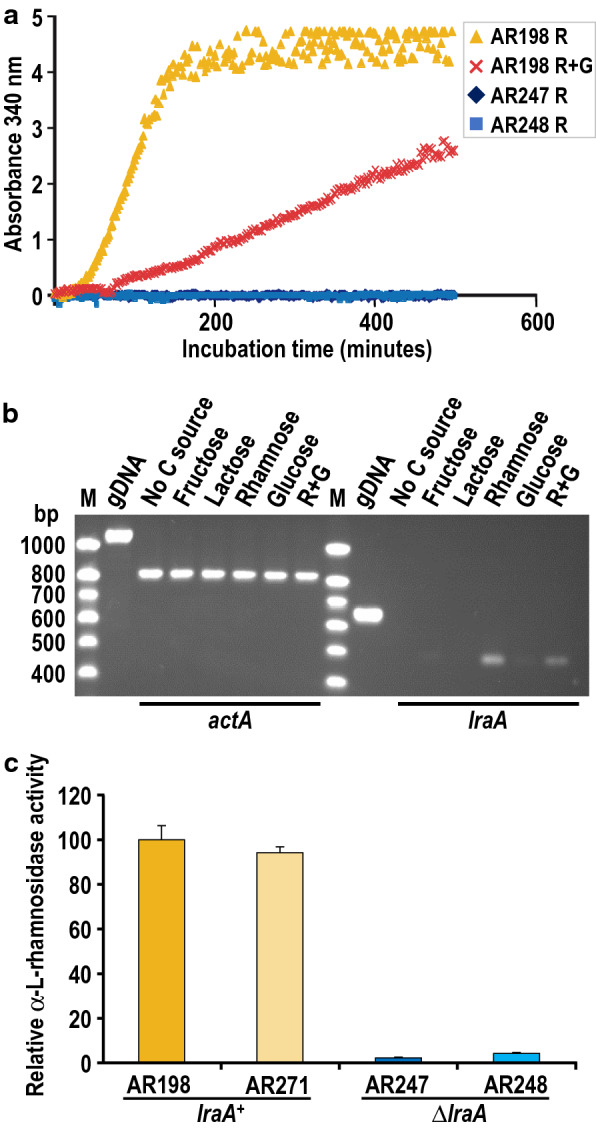
Physiological function of *lraA*/AN4186 in l-rhamnose catabolism. **a** l-Rhamnose dehydrogenase activity: increase in absorbance at 340 nm upon incubation of l-rhamnose with cell free extracts of untransformed *lraA*^+^ (AR198) and Δ*lraA* (AR247 and AR248) strains in the presence of NAD^+^ at 37 °C. **b** Expression of *lraA* in the *biA1* wild type control strain AR5 under different growth conditions: RT-sqPCR of RNA isolated from mycelia obtained 3 h after transfer to media lacking a carbon source (non-inducing/non-repressing conditions), 0.1% fructose (i.e. the pre-growth conditions), 2% lactose (non-inducing), 1% l-rhamnose (inducing), 1% glucose (repressing), and 1% l-rhamnose + 1% glucose (R + G inducing/repressing). The actin *actA* gene was used as a constitutive expression control for normalization. Amplifications (PCR) were reduced to 24 (*actA*) and 25 (*lraA*) cycles in order to obtain semi-quantitative data. PCR amplifications of gDNA are shown. M, low molecular weight marker (base pairs). **c** Deletion of *lraA*/AN4186 dramatically affects the production of α-l-rhamnosidase activity: extracellular α-l-rhamnosidase activities in *lraA*^+^ (AR198 and the isogenic nutritional control AR271) and Δ*lraA* (AR247 and AR248) strains 48 h after transfer to inducing conditions (1% l-rhamnose). Average activities and standard deviations of duplicates of two independent biological experiments are presented as percentages of those observed in AR198

To verify the function of the *lraA*/AN4186 gene product, the NAD^+^-dependent l-rhamnose-1-dehydrogenase (LraA) activity present in Δ*lraA* (AR247 and AR248) and *lraA*^+^ (AR198) cell-free extracts obtained from cultures transferred to l-rhamnose medium was assayed by direct NADH detection at 340 nm as described previously [11]. In contrast to AR198, NADH formation was not detected in the deletion mutant extracts upon addition of l-rhamnose (Fig. 3a) thus supporting the identification of AN4186 as the locus that encodes LraA. The data also reveal that under the experimental conditions no other *A. nidulans* enzyme compensates the lack of LraA activity in the ΔAN4186 mutants.

Glucose is known to repress l-rhamnose dehydrogenase activity in *A. pullulans* and *S. stipitis* [23, 24]. It was therefore of interest to study the kinetics of LraA activity in AR198 cultivated under inducing/repressing conditions (1% l-rhamnose + 1% glucose). Figure 3a shows that the rate of NADH formation was reduced in the presence of glucose hence the effect of glucose on the production of LraA activity in *A. nidulans* is similar to that observed in the earlier studies.

### LraA production is regulated at the level of transcription

In a first approximation to see whether l-rhamnose induction and carbon catabolite repression (CCR) of the synthesis of LraA in *A. nidulans*—and by extension the function of the LRA pathway—occurs at the level of transcription, semi-quantitative RT-sqPCR was performed revealing lower levels of *lraA* transcript accumulation under inducing/repressing compared to inducing conditions whilst transcript levels of the reference gene *actA* were similar in both. These data are consistent with glucose repression of LraA production at the level of transcription. That *lraA* transcripts were not detected or were much reduced in mycelia transferred to medium either lacking a carbon source or containing a carbon source other than l-rhamnose (Fig. 3b) indicates that transcriptional regulation of *lraA* occurs by specific induction in the presence of l-rhamnose rather than derepression.

The above genetic and biochemical analyses demonstrate the physiological relevance of the *lraA* gene in the utilization of l-rhamnose by *A. nidulans*. The inability of knockout strains (Δ*lraA*) to grow on l-rhamnose also provides convincing evidence that *A. nidulans* uses the non-phosphorylated pathway for l-rhamnose catabolism and that there are no alternative routes.

### Deletion of *lraA* impairs the production of α-l-rhamnosidases

It has been established previously that l-rhamnose induces the expression of the *A. nidulans* α-l-rhamnosidase genes at the level of transcription [12] and requires the presence of the Zn_2_Cys_6_ transcription activator RhaR [26]. To investigate whether LraA plays a role in this induction, extracellular α-l-rhamnosidase activity was measured in mycelia of *A. nidulans lraA*^+^ (i.e. AR198 and the nutritional control AR271) and *lraA*-deleted strains (AR247 and AR248) 48 h after transfer from 2% lactose MM to MM containing 1% l-rhamnose as sole carbon source. In contrast to the *lraA*^+^ strains α-l-rhamnosidase activity was drastically reduced in both of the Δ*lraA* mutants (< 5% of the level in wild type; Fig. 3c). Hence the *lraA* gene—and therefore the LRA pathway—plays a positive role in the biosynthesis of α-l-rhamnosidases (enzymes that liberate l-rhamnose from complex substrates).

These data indicate that the physiological inducer of α-l-rhamnosidase production in *A. nidulans* is in fact a product of the l-rhamnose catabolic pathway, thus making this route not only essential for the assimilation of this sugar but also the source of the inducer and hence having a role in regulating transcription.

### Re-introduction of *lraA* into the Δ*lraA* mutant restores the ability to grow on l-rhamnose and produce α-l-rhamnosidases

**Fig. 4:**
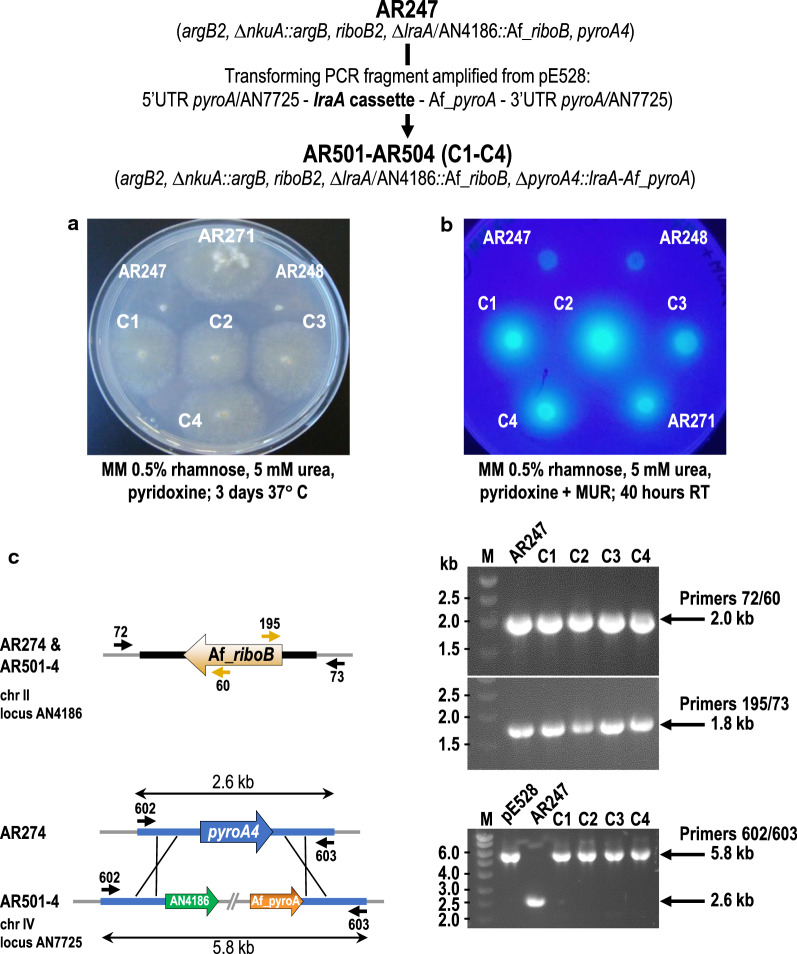
Ectopic expression of *lraA* in the Δ*lraA* mutant background restores both growth on l-rhamnose and α-l-rhamnosidase activity. Transformation-mediated ectopic replacement of *pyroA4* by functional *lraA* in AR247 (∆*lraA*). **a** Plate growth tests of the isogenic *lraA*^+^ nutritional control AR271, ∆*lraA* strains (AR247 and AR248) and four *lraA*^+^ complemented transformants (C1-C4) on l-rhamnose after 3 days at 37 °C. **b** MUR-mediated detection (under UV illumination) of α-l-rhamnosidase activity halos produced by *lraA-*complemented transformants (C1–C4) and control AR271 (*lraA*^+^) strains compared to ∆*lraA* (AR247 and AR248) after 40 h at RT. **c** Diagnostic PCRs confirm the absence of the *lraA* gene (AN4186) on chromosome II (replaced by Af_*riboB*) and replacement of the mutant *pyroA4* allele at locus AN7725 by the *lraA* expression cassette and Af_*pyroA*—i.e. substitution of the 2.6 kb DNA fragment in AR247 by the 5.8 kb complementation cassette in transformants C1–C4

To confirm that the Δ*lraA* mutant phenotype is specifically the result of *lraA* deletion, ectopic complementation at the *pyroA* (AN7725) locus was undertaken. An *lraA* complementation cassette (5′UTR *pyroA*/AN7725—*lraA* expression cassette—Af_*pyroA*—3′UTR *pyroA*/AN7725) generated by high-fidelity PCR from plasmid pE528 employing primers 602 and 603 (see “Materials and methods” for details) was used to transform AR247 (relevant genotype Δ*lraA*::Af_*riboB*, Δ*nkuA*::*argB*, *pyroA4*) and four pyridoxine prototrophic transformants (AR501-AR504) were selected and purified. The phenotypes of the latter were tested on solid medium containing l-rhamnose with and without the presence of the fluorogenic artificial substrate MUR: all four were able to grow on l-rhamnose (Fig. 4a) and also degrade MUR (Fig. 4b). As expected, the control strain AR271 (not deleted for *lraA*) was also able to grow on l-rhamnose and hydrolyse MUR whereas the Δ*lraA* mutants AR247 and AR248 were not. The *lraA* gene is thus able to restore the wild type phenotype as manifested by recovery of the ability to utilise l-rhamnose as a carbon source as well as the production of UV fluorescent halos resulting from MUR degradation by α-l-rhamnosidase activity. Diagnostic PCRs (Fig. 4c) confirmed the unaltered nature of the Δ*lraA*::Af_*riboB* deletion on chromosome II and ectopic insertion of the complementing cassette at the *pyroA* locus (AN7725 on chromosome IV) in all four selected transformants.

These results along with the previous data confirm the role of the *A. nidulans lraA* gene in the metabolism of l-rhamnose and the induction of α-l-rhamnosidase synthesis.

### *A. nidulans lra* gene expression is induced on l-rhamnose via RhaR and subject to carbon catabolite repression independently of CreA

Biochemical (LraA activity) and genetic (RT-sqPCR) analyses have suggested that *lraA* co-regulates with *rhaA* and *rhaE* (see Fig. 3a, b). Two modes of control of the expression of the *A. nidulans* α-l-rhamnosidase genes are known: specific induction in the presence of l-rhamnose which requires the function of the zinc binuclear cluster protein RhaR [12, 26], and carbon catabolite repression which involves an as yet undefined regulatory circuit different to that of the wide domain Cys_2_His_2_ repressor CreA [12]. We therefore sought to establish whether expression of the genes of the l-rhamnose catabolic pathway (LRA) is similarly controlled. Using appropriately designed primer pairs (Additional file 2: Table S1 and “Materials and methods”) RT-qPCR was undertaken to assess the relative abundances of transcripts of the *lraA*/AN4186, *lraB*/AN3740 and *lraC*/AN5672 genes, along with those of *rhaA* and *rhaE*, in total RNAs isolated from mycelia of three genetic backgrounds (wild type, Δ*rhaR* and *creA*^*d*^*30*) that were initially grown for 18 h on 0.1% fructose minimal medium (MM) and subsequently transferred for 3 h to MM supplemented with single or mixed carbon sources (1% l-rhamnose, 1% l-lactose, and a 1% l-rhamnose + 1% glucose mixture).

**Fig. 5:**
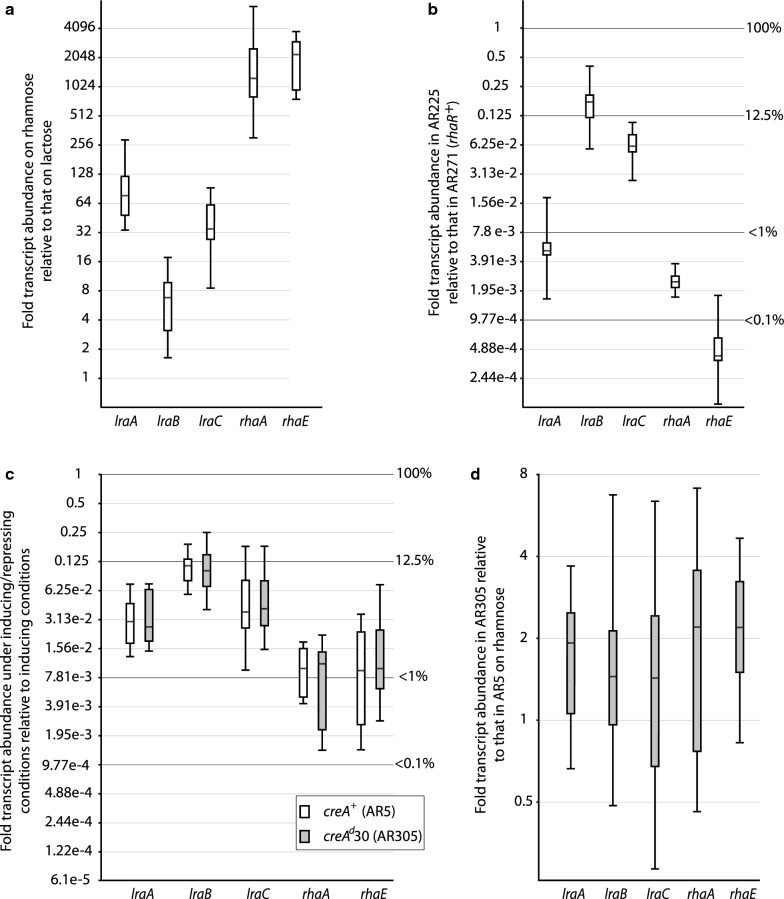
Analysis of *lra* and *rha* gene expression. Relative abundances of *lra* and *rha* transcripts: **a** in wild type mycelia (AR5) 3 h after transfer to MM containing l-rhamnose as sole carbon source compared to transfer to MM containing lactose; **b** in the *rhaR*-deletion strain (AR225) compared to the *rhaR*^+^ isogenic control (AR271) 3 h after transfer of each to MM containing l-rhamnose; **c** in the isogenic wild type (AR5; *creA*^+^—unshaded box) and *creA*^*d*^*30* (AR305; shaded box) strains 3 h after transfer to inducing/repressing conditions compared to the corresponding gene and strain 3 h after transfer to inducing conditions; **d** in *creA*^*d*^*30* compared to wild type 3 h after transfer of each to l-rhamnose-containing medium. Box and whisker plots were generated using the program REST-2009 (Qiagen). The box areas of the plots encompass 50% of all observations, the dotted line represents the sample median and each whisker represents the outer 25% of observations. The Cp data used in these analyses were obtained from three independent biological replicates and RT-qPCR was done using three technical replicates. The genes encoding histone H2B (AN3469) and beta-tubulin *benA* (AN1182) were used as references for normalisation of expression. Primer pairs used were: *lraA* 398/399; *lraB* 404/405; *lraC* 346/347; *rhaA* 410/411; *rhaE* 352/353; H2B 394/395; *benA* 386/387

Gene induction was examined by assessing the relative abundances of each of the transcripts in the wild type strain after 3 h in the presence of l-rhamnose compared to lactose. Box and whisker plots presented in Fig. 5a show that like *rhaA* and *rhaE*, transcript abundance of the three *lra* genes is greater on l-rhamnose, with *lraA* and *lraC* being induced approximately 80- and 35-fold, respectively; *lraB* transcript abundance also increased but only by a factor of about 6. Consistent with qualitative observations made previously by northern blotting [12], *rhaA* and *rhaE* are strongly induced on l-rhamnose yielding transcript abundances that are 3 orders of magnitude (i.e. > 1000-fold) greater than that on lactose. Expression of the *lraA*, *lraB* and *lraC* genes is thus a consequence of specific induction in the presence of l-rhamnose and not derepression, with *lraA* and *lraC* being more strongly induced than *lraB*. Expression analysis in *A. niger* also indicated a lower level of induction of *lraB* [22].

To investigate gene induction mediated by the transcriptional activator RhaR, transcript abundancies were examined in isogenic *rhaR*^+^ and *rhaR*-deleted (Δ*rhaR*) mycelia (strains AR271 and AR225, respectively) after their transfer to media containing l-rhamnose (inducing conditions) as sole carbon source. As can be seen in Fig. 5b very low abundance ratios (< 1%) were found for *lraA*, *rhaA* and *rhaE* transcripts thus reflecting considerably reduced expression (2–3 orders of magnitude) of the corresponding genes in the deletion strain. Indeed, the Cp values recorded for them in the deletion strain were around the limits of detection (Cp 30–35). Similarly to observations in *A. niger* [22], *lraB* is the gene least influenced by RhaR. These observations confirm the role of RhaR in mediating induction of the LRA pathway in *A. nidulans*.

Our data have indicated that the physiological inducer of rhamnosidase synthesis is a product of the LRA pathway (Fig. 3c) rather than l-rhamnose itself. CCR of LRA reactions preceding production of the inducer would thus lead to a reduced concentration or absence of the latter and therefore a negative effect on target gene induction. To investigate the possible influence of CCR and CreA on the pathway, RT-qPCR was carried out on RNAs isolated from mycelia of the *A. nidulans* wild type *creA*^+^ (AR5) and the strongly derepressed *creA*^*d*^*30* mutant (AR305) after transfer to inducing (l-rhamnose) and inducing/repressing (l-rhamnose + glucose) conditions. As can been seen from the plots in Fig. 5c expression of the *lraA*, *lraB* and *lraC* genes in the *creA*^+^ strain is repressed in inducing/repressing medium and this is effected in a manner independent of CreA since this repression is also seen in the *creA*^*d*^*30* genetic background. Indeed, the degree of glucose repression of each of the genes is similar in both the wild type and *creA*^*d*^*30* strains. Additionally, the RT-qPCR results are in agreement with our earlier northern blot data for *rhaA* and *rhaE* [12].

Taken together, the above RT-qPCR data show that both the induction and repression profiles of the three LRA pathway genes analysed closely parallel those of the rhamnosidase genes *rhaA* and *rhaE*. To the best of our knowledge this is the first demonstration of CreA-independent CCR operating on a plant cell wall sugar catabolic pathway in fungi.

Finally, it is also noteworthy that under inducing (1% l-rhamnose) conditions transcript abundancies of the three catabolic genes were found to be somewhat elevated in the derepressed *creA*^*d*^*30* strain compared to the wild type (Fig. 5d), an effect observed earlier in northern blot analyses of *rhaA* and *rhaE* [12]. This indicates a degree of repression by CreA on each of the five genes under inducing conditions.

### Deletion of *lraA*/AN4186 negatively affects the expression of l-rhamnose‐inducible genes

**Fig. 6:**
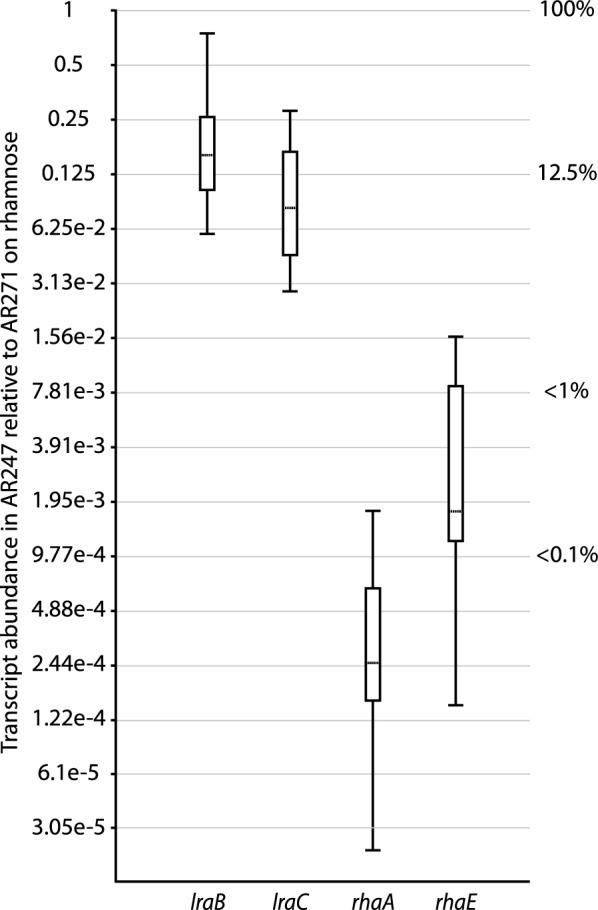
Analysis of *lra* and *rha* gene expression in the *lraA* deletion mutant under inducing conditions. Relative transcript abundances in ∆*lraA* (AR247) compared to the isogenic *lraA*^+^ control (AR271) 3 h after transfer of each to l-rhamnose-containing medium. The Cp data were obtained from two independent biological replicates and qPCR was done on three technical replicates. Primer pairs and normalization were as noted in Fig. 5

To corroborate the influence of the LRA pathway on the expression of those genes induced in the presence of l-rhamnose via RhaR we examined transcript abundances in the Δ*lraA* mutant (AR247) relative to those of the isogenic riboflavin nutritional control AR271 (*lraA*^+^) under inducing conditions. As can be seen in Fig. 6, relative transcript levels of the genes of the catabolic pathway as well as those encoding the rhamnosidases were reduced in the absence of a functional pathway—in a manner approximately inversely proportional to the RhaR-mediated induction of each on l-rhamnose—thus showing the importance of the pathway for induction.

## Conclusions

In summary, *A. nidulans* genes (*lraA*, *lraB* and *lraC*) that are candidates for encoding the first three l-rhamnose catabolic activities of the phosphorylated intermediate (LRA) pathway have been identified by virtue of sequence homologies shared with the products of previously characterized *LRA* genes in *S. stipitis* [11, 20]. The phenotype of *lraA/*AN4186 deletion mutants has demonstrated the importance of the LRA pathway for both the utilization of l-rhamnose and the induction of rhamnosidase genes. Data from expression analyses are consistent with the involvement of the *lraB* and *lraC* genes, as well as *lraA*, in the l-rhamnose catabolic pathway, and have also revealed the novelty that CCR of the LRA pathway is mediated by an as yet unknown non-CreA mechanism.

## Supplementary information

**Additional file 1: Figure S1.** Schemes of microbial l-rhamnose catabolic pathways. (A) Catabolic pathways for l-rhamnose and (B) organisation of the genes encoding the corresponding activities in *E. coli* and various fungi. Homologous genes are indicated using the same colour code. Chr: chromosome number.

**Additional file 2: Table S1.** Oligonucleotide primers used in this study.

## Data Availability

All data generated or analysed during this study are included in this published article and its additional files. The *A. nidulans* cDNA sequence of *lraA* has been deposited with GenBank (Accession number: MT431619).
