# Correction to: Catabolism of l-rhamnose in *A. nidulans* proceeds via the non-phosphorylated pathway and is glucose repressed by a CreA-independent mechanism

**DOI:** 10.1186/s12934-021-01708-x

**Published:** 2021-12-07

**Authors:** Andrew P. MacCabe, Elpinickie I. Ninou, Ester Pardo, Margarita Orejas

**Affiliations:** 1 Instituto de Agroquímica y Tecnología de Alimentos (IATA), Consejo Superior de Investigaciones Científcas (CSIC), c/Catedrático Agustín Escardino Benlloch 7, 46980 Paterna, Valencia Spain; 2 Present Address: Center for Basic Research, Biomedical Research Foundation, Academy of Athens (BRFAA), 4 Soranou Ephessiou Street, 11527 Athens, Greece; 3 Present Address: ADM Biopolis, Parque Científco Universidad de Valencia, c/Catedrático Agustín Escardino Benlloch 9, 46980 Paterna, Valencia Spain

## Correction to: Microb Cell Fact (2020) 19:188 https://doi.org/10.1186/s12934-020-01443

Following publication of the original article [1], the authors identified an error in funding section. The correct funding note is given in this Correction article.

**Funding:** This work was supported by the Spanish Ministerio de Ciencia e Innovación/FEDER and Ministerio de Economía y Competitividad/FEDER (Grant Numbers AGL2011-29,925 and AGL2015-66,131-C2-2-R, respectively). The original article has been revised.
