# Supplementary figures and images for: Catabolism of l-rhamnose in A. nidulans proceeds via the non-phosphorylated pathway and is glucose repressed by a CreA-independent mechanism
Source: Microb Cell Fact. 2020 Oct 2;19:188. doi: 10.1186/s12934-020-01443-9 (PMC7532622; doi:10.1186/s12934-020-01443-9)

**A**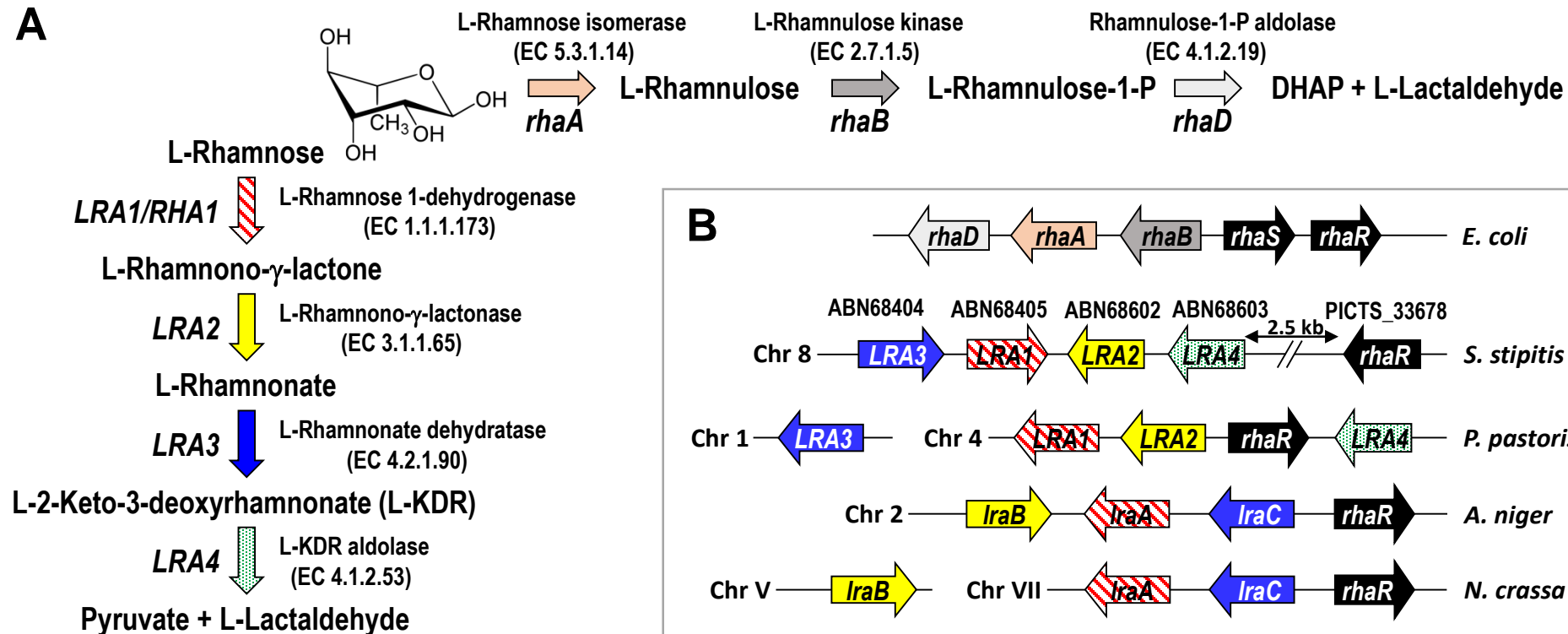**B**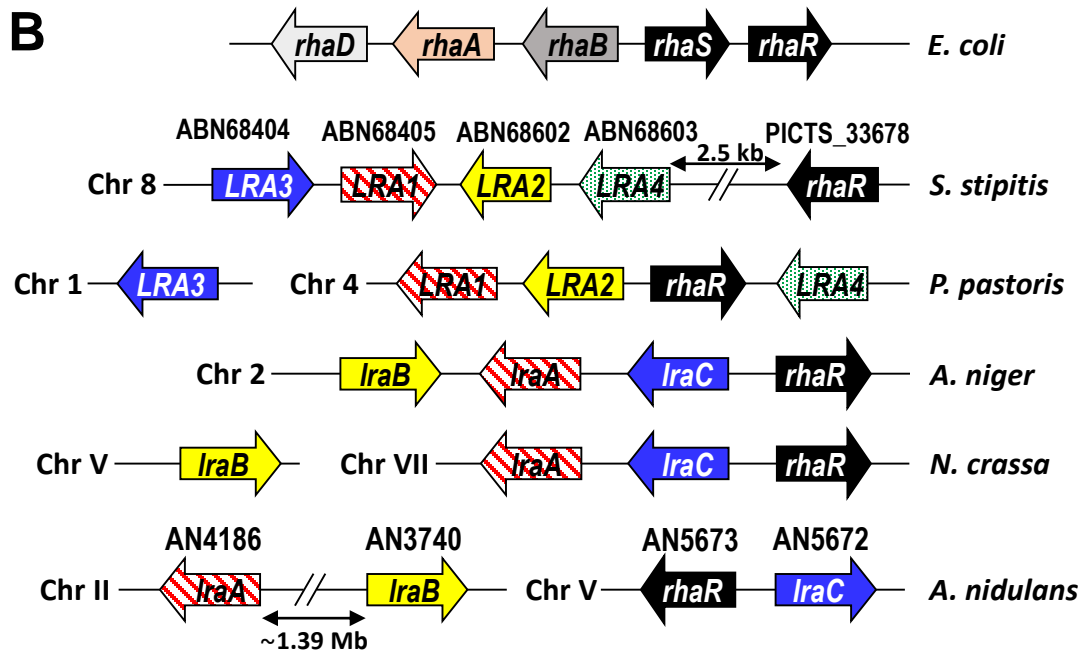

Figure S1

Supplement: Supplementary file 1 — Additional file 1: Figure S1. Schemes of microbial l-rhamnose catabolic pathways. (A) Catabolic pathways for l-rhamnose and (B) organisation of the genes encoding the corresponding activities in E. coli and various fungi. Homologous genes are indicated using the same colour code. Chr: chromosome number. [file 12934_2020_1443_MOESM1_ESM.pdf]
